# Defining the lateral edge of the femoroacetabular articulation: correlation analysis between radiographs and computed tomography

**DOI:** 10.1007/s11832-016-0768-y

**Published:** 2016-08-19

**Authors:** Ashish Mittal, James D. Bomar, Megan E. Jeffords, Ming-Tung Huang, Dennis R. Wenger, Vidyadhar V. Upasani

**Affiliations:** 1 Department of Orthopedics, University of California, San Diego, San Diego, CA USA; 2 Department of Orthopedics, Rady Children’s Hospital, San Diego, 3030 Children’s Way, Suite 410, San Diego, CA 92123 USA; 3 National Cheng Kung University Hospital, Tainan City, Taiwan

**Keywords:** LCEA, Lateral center edge angle, Sourcil, Hip dysplasia

## Abstract

**Purpose:**

The purpose of this study was to analyze the variation in measuring the lateral center edge angle of Wiberg (LCEA) using the lateral edge of the sourcil (LCEA-S) compared to the lateral edge of the acetabulum (LCEA-E), and to correlate these measurements with three-dimensional computed tomography (3D-CT)-based analysis of the femoroacetabular articulation.

**Methods:**

A retrospective analysis was performed on 24 patients (45 hips) treated for hip dysplasia at a single institution. All patients were required to have an anteroposterior (AP) pelvis radiograph and pelvic CT. LCEA-S and LCEA-E measurements were calculated from radiographs. Axial CT images were processed to standardize pelvic orientation and calculate the LCEA at three points (posterior, central, anterior) along the acetabular edge. Correlation analysis was used to evaluate radiographic and CT measures.

**Results:**

Eight males and 16 females with an average age of 14.6 years were included. The LCEA-S (16.5° ± 2.0°) was found to be significantly less than the LCEA-E (26.0° ± 2.0°) (*p* < 0.001). The LCEA-S had the greatest correlation with the central measurement on the 3D-CT (*r*_s_ = 0.893; *p* < 0.001). The LCEA-E had the greatest correlation with the anterior measurement on the 3D-CT (*r* = 0.834; *p* < 0.001).

**Conclusions:**

The LCEA can change significantly depending on the bony landmark used to define the lateral edge of the femoroacetabular articulation. The edge of the sourcil most closely correlates with the central weight-bearing portion of the articular surface on the 3D-CT and should be used to define the LCEA when treating patients with hip dysplasia.

**Level of evidence:**

Level III, retrospective comparison study.

## Introduction

Developmental dysplasia of the hip is a disorder of infancy that affects approximately 3–4 children per 1000 live births in the United States [1]. Unidentified and untreated developmental dysplasia can lead to serious consequences, such as premature osteoarthritis [2–4]. Radiographs play a practical and indispensable role in the diagnosis and management of hip dysplasia due to their low cost and modest radiation exposure [5–8] [as compared to computed tomography (CT) studies].

**Fig. 1:**
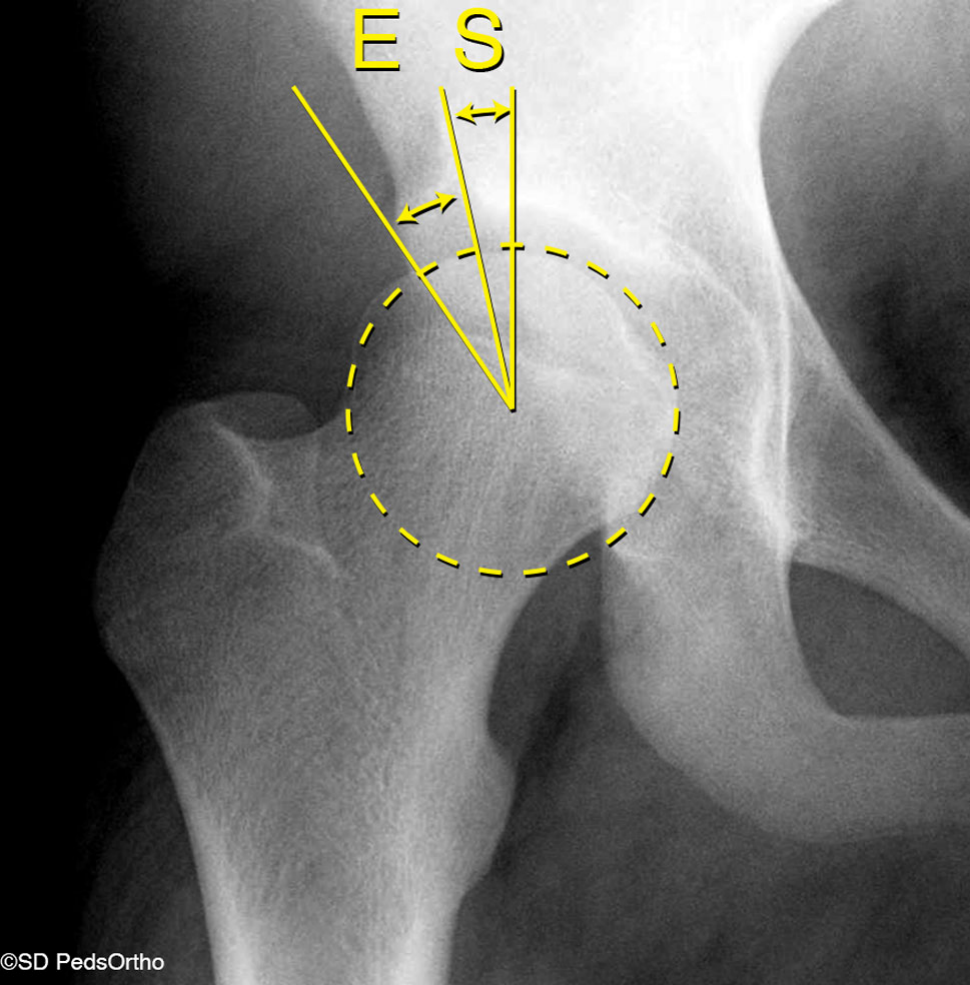
Anteroposterior X-ray of the right hip indicating the method of measurement for the lateral center edge angle measured to the edge of the acetabulum, as described by Wiberg (*E*) and to the edge of the sourcil (*S*)

Various radiographic measurements including the center edge angle of Wiberg or lateral center edge angle (LCEA), the acetabular index (AI), and the anterior center edge angle (ACEA) have been proposed to aid with the diagnosis of developmental dysplasia [3, 9]. In particular, the LCEA and ACEA have been shown to be linked to early-onset osteoarthritis in cases of hip dysplasia [10]. The LCEA traditionally represents lateral coverage of the femoral head by the acetabulum. It was defined by Wiberg (as the center edge angle) in 1939 as the angle formed between a line running through the center of the femoral head parallel to the body and a line drawn from the center of the femoral head to the lateral edge of the acetabular roof [3] (Fig. 1).

Several decades later, Ogata et al. proposed a “refined” LCEA measured to the edge of the sourcil (LCEA-S) (Fig. 1), as opposed to the bony edge of the acetabulum (LCEA-E), to more accurately diagnose hip dysplasia [11]. However, identification and accurate measurement to the edge of the sourcil may be challenging, especially in younger individuals with hip dysplasia, leading to higher rates of interobserver agreement for measures to the lateral acetabular edge [12, 13]. Both techniques continue to be used today, and there are differing opinions on which is the most accurate and precise.

Therefore, the purpose of this study was to compare LCEA-S and LCEA-E measurements and correlate these values to LCEA values as measured on anterior, central, and posterior planes in 3D constructed models using CT. Our objective was to better elucidate how these measurements differ from one another and if these differences could be accounted for using 3D reconstructions. We hypothesized that LCEA measurements to the lateral edge of the sourcil on the anteroposterior (AP) radiograph would more closely correlate with the weight-bearing surface on the 3D reconstructed models of the pelvis.

## Methods

After Institutional Review Board (IRB) approval was obtained, a retrospective review of radiographic and CT imaging on 24 patients (45 hips) aged 10–20 years treated for hip dysplasia at a single institution between June 2008 and August 2014 was performed. Skeletally immature patients were excluded if they did not have a pelvic CT scan within 4 months of an AP pelvis radiograph. Skeletally mature patients were excluded if they did not have a pelvic CT scan within 2 years of an AP pelvis radiograph. Patient charts were reviewed for demographic data, including age, gender, and diagnosis.

Pelvis radiographs were used for calculation of the LCEA to the edge of the acetabulum and to the edge of the sourcil, and for the presence or absence of subluxation (Fig. 1). The center of the femoral head was first obtained using a best-fit circle. The center of both femoral heads was then connected as a reference for the horizontal axis. The lateral edge of the acetabulum was defined as the lateral most ossification. The lateral edge of the sourcil was defined as the most lateral end of the sclerotic subchondral bone. The LCEA was then calculated by a line perpendicular to the horizontal axis and a line from the femoral head center to the lateral edge of the sourcil for LCEA-S and to the lateral edge of the acetabulum for LCEA-E. Calculation of the LCEA-S and LCEA-E measurements were made by two independent fellowship trained, orthopedic surgeons with less than 5 years of clinical practice to determine inter- and intrarater reliability. The reviewers were blinded to the measurements of each other and to the identity of patients. All measurements were made on digital radiographs using Merge PACS measurement tools (version 6.5.6; Merge Healthcare, Chicago, IL, USA).

**Fig. 2:**
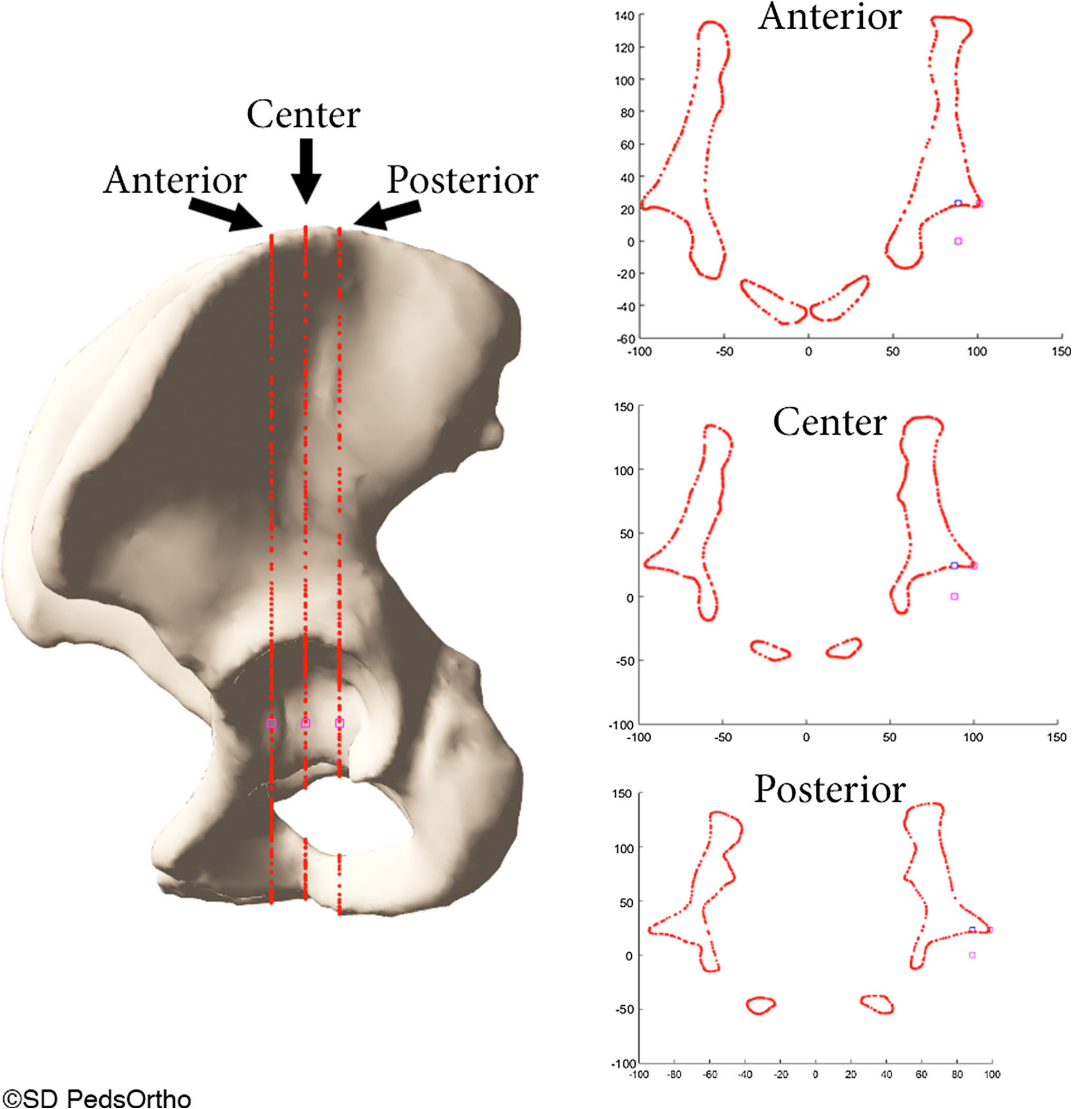
Diagram indicating the location of measurement (anterior, center, posterior) for the center edge angle measured on computed tomography

A 3D reconstruction of each pelvis was created from CT data using Mimics software (Materialise, Leuven, Belgium). Custom MATLAB software (MathWorks, Natick, MA, USA) was used to standardize pelvic orientation to account for tilt and rotation, as previously described by our institution [14]. Acetabulum surfaces were automatically identified on the 3D pelvic models and fitted with a best-fit sphere using least-squares regression [15]. The LCEA was calculated on a coronal slice defined by the center of the best-fit sphere of the acetabulum to points posterior or anterior along the edge of the acetabulum, and a normal vector to the slice plane (Fig. 2). Posterior and anterior points along the acetabulum were identified based on a central angle of 22.5° from the acetabulum center, representing an overall weight-bearing surface arc of 45°.

Basic descriptive statistics are reported. The Shapiro–Wilk test of normality was used on all data. The distribution of LCEA-S was found to be non-normal. The Mann–Whitney test was used to evaluate differences in LCEA-S and LCEA-E. Pearson correlation (*r*) or Spearman’s rho (*r*_s_) was used to evaluate correlations among radiographic and CT data. Statistical significance was defined as *p* < 0.05. All statistical analysis was conducted using SPSS (version 12; SPSS, Chicago, IL, USA).

## Results

Eight males and 16 females with an average age of 14.6 years (range 10.4–19.8) were included in the study. The intra-class correlation coefficient (ICC) among the two observers was found to be 0.951 for LCEA-S and 0.855 for LCEA-E (*p* < 0.001). As expected, the mean LCEA-S [16.5° ± 2.0°; 95 % confidence interval (CI) = 12.4–20.5] was found to be significantly less than the mean LCEA-E (26.0° ± 1.7°; 95 % CI = 22.6–29.4) (*p* < 0.001) on AP pelvis radiographs.

LCEA measurements were obtained for anterior (mean 23.1° ± 1.7; 95 % CI = 19.7–26.5), central (mean 23.5° ± 1.5; 95 % CI = 20.5–26.5), and posterior (mean 21.4° ± 1.6; 95 % CI = 18.2–24.5) coronal planes on the 3D reconstructions.

**Fig. 3:**
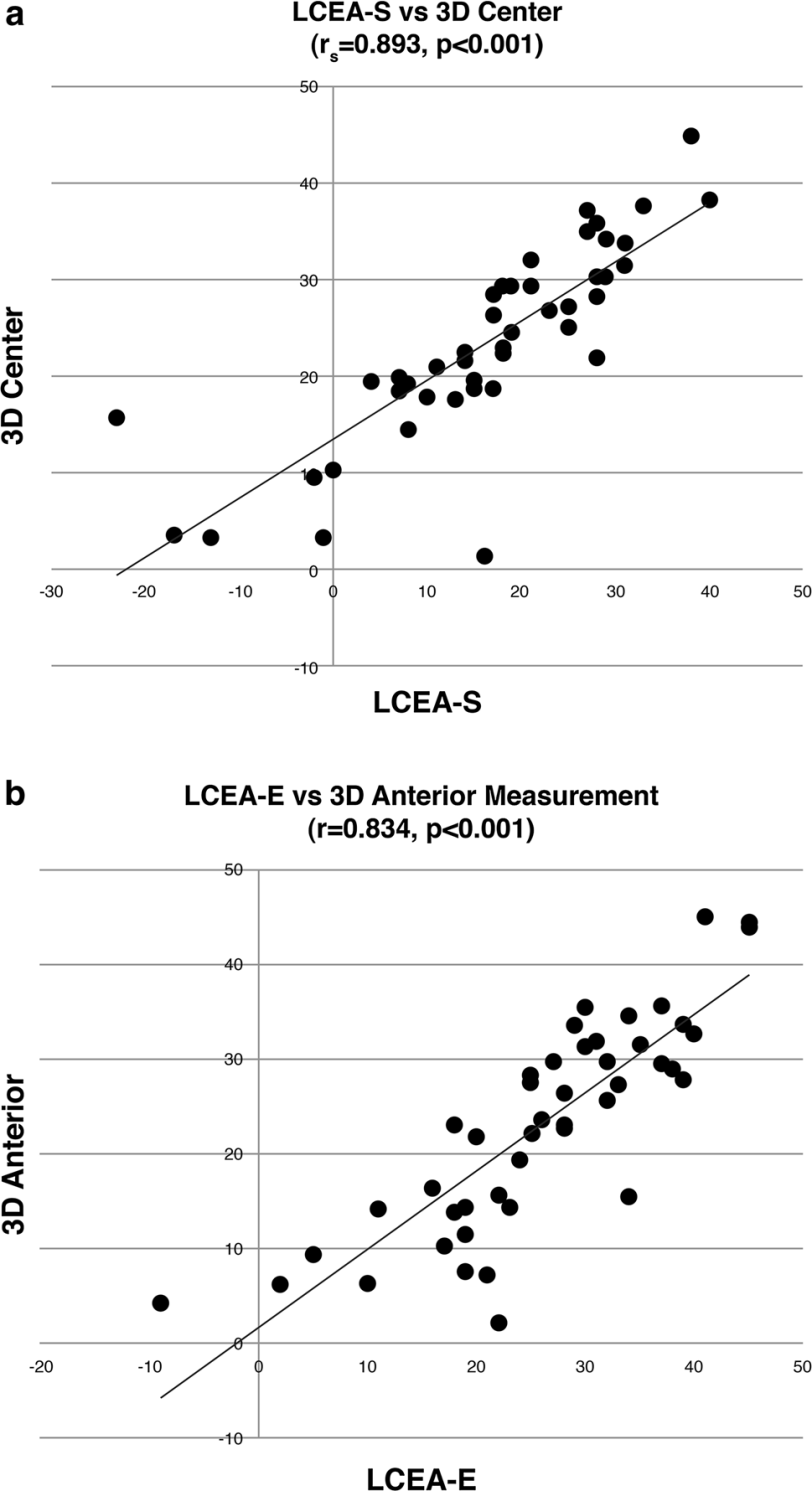
**a** Scatter plot illustrating the correlation between the lateral center edge angle measured at the sourcil on X-ray and the measurement made at the center of the acetabulum on computed tomography. **b** Scatter plot illustrating the correlation between the lateral center edge angle measured at the edge of the acetabulum on X-ray and the measurement made at the anterior location on computed tomography

The LCEA-S correlated most strongly with LCEA measurements in the central plane of 3D constructed models (*r*_s_ = 0.893; *p* < 0.001) (Fig. 3a). Correlations in the anterior and posterior CT planes were *r*_s_ = 0.868 and *r*_s_ = 0.654, respectively (*p* < 0.001). The LCEA-E values correlated most strongly with CT measurements in the anterior plane (*r* = 0.834; *p* < 0.001) (Fig. 3b), with diminished correlation in the central and posterior planes (*r* = 0.784 and 0.587; *p* < 0.001). Correlation between LCEA-S and LCEA-E values was *r*_s_ = 0.863 (*p* < 0.001).

## Discussion

The LCEA (originally described by Wiberg as the center edge angle) is an important radiographic parameter in diagnosing hip dysplasia, as it represents superolateral coverage of the femoral head in the coronal plane on AP radiographs [3, 9, 11–14]. Undiagnosed or incompletely treated hip dysplasia can lead to serious consequences, including premature osteoarthritis [2–4]. While the sourcil more accurately represents the standard lateral edge of the weight-bearing acetabulum, it has been debated whether this angle should be measured to the lateral edge of the acetabulum or to the lateral edge of the sourcil [11, 12]. These two measurements have been shown to significantly differ from one another, especially in younger children, cases of acetabular retroversion, and low anterior inferior iliac spine (AIIS), leading to errors in diagnosis [11–13, 16–18]. In this study, we confirm that there is, on average, a 10° difference between these two measurements. In addition, we were able to show that the LCEA-S better correlates with the central weight-bearing portion of the articular surface on 3D analysis and is, thus, a superior indicator of femoral head coverage in hip dysplasia.

There have been various conclusions that have been drawn regarding the correlation of the LCEA on AP radiographs to 3D imaging in the past several years. Ogata et al. showed that the LCEA-S better correlated with acetabular head coverage on transverse CT than the LCEA-E [11]. It has also been suggested using CT and magnetic resonance imaging (MRI) that the lateral bony margin of the acetabulum represents the anterolateral margin of the acetabulum, whereas the lateral end of the sourcil corresponds to the lateral edge of the mid-superior acetabulum [12]. Additionally, LCEA measurements on AP radiographs using a single technique have been shown to correlate strongly to LCEA measurements on CT [5, 14, 19, 20]. Stelzeneder et al. compared LCEA-E measurements using the classic approach from Wiberg to measurements on different planes of anterior (10 mm), anterior (5 mm), central, and posterior (5 mm) on MRI and found that the LCEA-E on radiographs correlated most closely with the 10 mm anterior measurement on MRI [21]. To our knowledge, however, this is the first study to directly compare the two different measurements of the LCEA to measurements on a 3D reconstruction of the pelvis on different coronal planes.

3D imaging is often used in conjunction with LCEA measurements on plain radiographs in the diagnosis and management of hip dysplasia. 3D CT provides advantages in the visualization of femoral head and acetabular structure and coverage that may be useful in diagnosis, preoperative planning, and postoperative assessment [7, 22]. It may also be useful in cases of acetabular retroversion, where the LCEA may underestimate femoral head coverage [20]. Both CT and X-ray are inadequate for measurement of the cartilaginous center edge angle, which has been shown to be an effective tool in characterizing femoroacetabular articulation [23]. Cost of use and radiation exposure with CT studies limit the use of these imaging modalities, but they still play an important role in diagnosis, operative planning, and postoperative management [5–8].

Methods have been developed to quantify the percentage acetabular coverage of the femoral head around the acetabular rim. MRI provides the added benefit of cartilage and soft tissue visualization, which can help greatly in identifying residual dysplasia where evidence is not visible on plain radiographs [24–26]. A few studies have quantitatively looked at acetabular index values of bony, cartilaginous, and labral coverage on MRI after closed and open reduction of the hip to identify values consistent with residual dysplasia [25–27]. Range of motion MRI is a useful tool that can be used to detect uncorrected labrum deformities and shifts in the zone of compressive force with abduction of the hip that can suggest suboptimal reduction in hip dysplasia [28]. The cost of MRI studies makes them impractical in most medical communities.

Understanding acetabular coverage and femoral head/acetabular relationships remain critical for assessing residual hip dysplasia in childhood. New technology offers new ways to assess traditional radiographic-based data. Correlation between traditional values, such as the CE angle, which had been established over several generations with a very large database (Tönnis and others) remain our standard reference [29]. All such values utilized the LCEA-E for determining normalcy regarding hip dysplasia.

It is becoming apparent that the LCEA-S offers a more accurate determination of femoral head coverage by the true articular surface of the acetabulum, making it a more accurate tool for identifying and treating functional dysplasia of the hip. As with any study involving radiographic measurement by multiple individuals, our study was susceptible to inter- and intraobserver errors in measurement. At this point, establishing exact values for normalcy vs. inadequate femoral head coverage relies on small sample size studies such as ours. Larger studies that include longitudinal follow-up of patients will be required before exact LCEA-S-based measurements can be used for treatment decisions. For example, a CE angle greater than 20° has traditionally suggested a good prognosis for hip dysplasia. We do not yet have data to know whether this same value (20°) should be used in determining a good prognosis for the hip.

## Conclusion

Frontal plane pelvis radiographs remain the standard protocol for diagnosing and assessing hip dysplasia in pediatric centers around the world [1]. The lateral center edge angle (LCEA) is a calculated angle used to define lateral coverage of the femoral head by the acetabulum, and is important in the diagnosis and management of hip dysplasia. Our 3D analysis shows that the edge of the sourcil most closely correlates with the central weight-bearing portion of the articular surface and, thus, should be used to define the LCEA and acetabular slope when treating patients with hip dysplasia.
